# Celiac—the lone horse? An autoimmune condition without signals of microbiota dysbiosis

**DOI:** 10.1128/spectrum.01463-23

**Published:** 2023-08-11

**Authors:** Sondra Turjeman, Efrat Sharon, Rachel Levin, Beata Oralewska, Anna Szaflarska-Popławska, Joanna B. Bierła, Bożena Cukrowska, Omry Koren

**Affiliations:** 1 Azrieli Faculty of Medicine, Bar-Ilan University, Safed, Israel; 2 Department of Gastroenterology, Hepatology, Nutritional Disorders and Pediatrics, The Children’s Memorial Health Institute, Warsaw, Poland; 3 Department of Pediatric Endoscopy and Gastrointestinal Function Testing, Ludwik Rydygier Collegium Medicum in Bydgoszcz, Nicolaus Copernicus University in Torun, Bydgoszcz, Poland; 4 Department of Pathomorphology, The Children’s Memorial Health Institute, Warsaw, Poland; Lerner Research Institute, Cleveland, Ohio, USA

**Keywords:** microbiome, celiac disease, diet

## Abstract

Accumulating evidence supports the role of microbiota in autoimmune processes, but research regarding the role of the gut microbiota in celiac disease (CD) is still emerging, and a consistent CD-associated dysbiosis pattern has not yet been defined. Here, we characterized the microbiota of children newly diagnosed with CD, with their unaffected family members as a healthy control group to reduce confounding factors including genetic background, hygiene, dietary habits, and environment, and followed children with CD over 1 year of dietary intervention (exclusion of gluten) to understand if the microbiota is associated with CD and its mediation. We did not find differences in the microbiota of siblings with and without CD, despite a wealth of evidence in the literature supporting CD-specific microbiota. CD is common among first-degree relatives, so this could suggest that unaffected family members in this study may be living in a pre-CD state, currently below clinical detection. Interestingly, despite the effectiveness of diet in CD control, we did not observe diet-mediated microbiota changes, except for short-term increase in *Akkermansia muciniphila*. This lack of effect could suggest a very strong CD microbial signature even when controlled or could be a technical shortcoming. Expanded future studies with both related and unrelated controls and diet interventions in both the CD and control arms can provide further context to our findings.

**IMPORTANCE:**

The microbiota is the community of microbes that live in and on us. These microbes are essential to our health and everyday function. Disruption of the community is associated with diseases ranging from metabolic syndrome to autoimmune diseases to mental disorders. In the case of celiac disease (CD), research remains inconclusive regarding implications of the microbiota in etiology. Here, we compared microbiota of children with CD to those of their unaffected family members and found very few differences in microbiota profiles. We next examined how gluten elimination in CD patients affects the microbiota. Surprisingly, despite diet adherence, microbiota shifts were minimal, with only a short-term increase in *Akkermansia muciniphila*. Previous studies suggest that family members of CD patients may be living in a pre-CD state, which could explain their microbial similarity. A larger study with unrelated controls and increased microbiota monitoring during diet intervention should give our findings more perspective.

## INTRODUCTION

Celiac disease (CD) is an autoimmune disorder in which consumption of gluten-containing foods results in inflammation and damage to the small intestine due to an off-target immune response. This typically results in a range of gastrointestinal symptoms as well as other complications including anemia, reduced bone density, and infertility (1). Besides immune and genetic factors, environmental factors seem to also play a role in the onset of CD (1), and presently, the recommended treatment is lifelong elimination of gluten from the diet.

It is suggested that the disturbances in the gut microbiota may be important in activation of autoimmune processes, and intestinal dysbiosis is known to be associated with a number of autoimmune conditions (2), including lupus (3, 4), autoimmune hepatitis (5), and multiple sclerosis (6), and gastrointestinal conditions including inflammatory bowel diseases (7 – 9). Research regarding the role of the gut microbiota in CD is now emerging, and bacterial differences between CD patients and healthy controls have been observed [reviewed in reference (10)], but a consistent dysbiosis pattern has not yet been defined. Furthermore, it is still unclear if microbial shifts are a cause or a result of CD or even an expression of a gluten-free diet–CD interaction.

In this study, we characterized the microbiota of children newly diagnosed with CD and of their unaffected family members. Using unaffected family members as a healthy control group reduces confounding factors including genetic background, hygiene, dietary habits, and environmental factors (e.g., pets, dust exposure). We also followed children with CD over 1 year of dietary intervention (exclusion of gluten) to understand if the microbiota is associated with CD and its mediation.

## MATERIALS AND METHODS

### Cohort recruitment and study design

The study included Polish families who were recruited at the Children’s Memorial Health Center, Warsaw, Poland and Ludwik Rydygier Collegium Medicum, Bydgoszcz, Poland, at the time of CD diagnosis of a child in the family. The inclusion criterion for recruitment was CD diagnosis according to the European Society for Pediatric Gastroenterology Hepatology and Nutrition (ESPGHAN) (11, 12), and those with potential CD, CD not diagnosed with ESPGHAN guidelines, or with gluten-free diet in any family members were excluded. No participants had received antibiotics or non-steroidal anti-inflammatory drugs at the time of recruitment or further sampling, nor did they suffer from acute infections. In total, nine families were recruited, each with one focal CD child, a mother, a father, and one or more siblings without a CD diagnosis (Table 1), living together in the same household. After diagnosis, the CD child was introduced to a gluten-free diet. Fecal samples were collected from each family member before starting dietary treatment (time 1, T1), and all family members were also screened for CD at the time of inclusion in the study. Additionally, after 6 (T2) and 12 months (T3), fecal and blood samples were collected from six CD children to assess concentrations of specific celiac antibodies against tissue transglutaminase (tTG) and characterize microbiota; other patients were lost to follow-up. Physical characteristics (viz. weight and height) were compared for focal children and siblings using Stata version 12 (13).

**TABLE 1:** Cohort characterization^*f*^

Characteristics	Celiac child (*n* = 9)	Siblings (*n* = 14)	Parents	
			Mothers^*a*^ (*n* = 9)	Fathers (*n* = 9)
Age (years): mean ± SD^*d*^	8.4 ± 2.0	9.1 ± 6.6	39.3 ± 5.8	40.5 ± 5.0
Range	4.7–10.7	1.8–22.6^*b*^	32.1–49.7	33.1–49.9
Number of males (%)	2 (22.2%)	5 (35.7%)	0	9 (100%)
Weight (kg): mean ± SD	30.1 ± 15.0	30.3 ± 16.6	60.8 ± 10.2	88.6 ± 20.2
Range	12.0–57.0	10.0–53.0	53.0–78.0	59.0–110.0
<3 Percentile: *n* (%)	1 (11.1%)	1 (9.1%)^*c*^	NA^*e*^	NA
>97 Percentile: *n* (%)	0	0	NA	NA
Height (cm): mean ± SD	127.3 ± 20,8	124.7 ± 24.8	158.6 ± 9.9	177.4 ± 3.3
Range	100.0–159.0	80.0–162.0	143.0–169.0	173.0–182.0
<3 Percentile *n* (%)	3 (33.3%)	1 (9.1%)^*c*^	NA	NA
>97 Percentile: *n* (%)	0	0	NA	NA

^*a*^ One mother was excluded from analyses as she was diagnosed with CD when her child was recruited to the study.

^*b*^ Three sisters were over the age of 18 years.

^*c*^ Only siblings ≤18 years of age included.

^*d*^ SD, standard deviation.

^*e*^ NA, not applicable.

^*f*^ Physical development (body weight and height) was assessed according to centile charts for the population of Polish children (OLAF national research project reference charts) (14). No differences were found in physical development between celiac disease (CD) children and their siblings.

The study was approved by the local Bioethical Committee of the Children’s Memorial Health Institute (no. 62/KBE/2016 dated 12 December 2016) and written informed consent was obtained from patients’ parents, caretakers, or patients over 16 years old concerning the use of their serum samples for scientific purposes. The study protocol conforms to the ethical guidelines of the 1975 Declaration of Helsinki.

### CD diagnosis and screening

CD was diagnosed according to ESPGHAN guidelines (11, 12) in subjects with positive anti-tTG antibodies of immunoglobulin A (anti-tTG-IgA) or anti-tTG-IgG in cases of IgA deficiency, and histological changes in duodenal biopsies described at least as Marsh 2. In three out of nine children with high concentrations of anti-tTG-IgA (>100 AU/mL, e.g., 10 times higher than the upper normal limit), positive anti-endomysial antibodies and haplotype HLA (human leukocyte antigen)-DQ2 and/or HLA-DQ8, CD was diagnosed without intestinal biopsy. Serological screening for CD was performed in all family members and contributed to CD detection in one mother (removed from microbiota analyses). The screening involved the measurement of anti-tTG-IgA and anti-tTG-IgG antibodies. The tests were performed with the use of a Phadia 100 analyzer and EliA commercial kits (Thermo Scientific Phadia GmbH, Freiburg, Germany).

### CD dietary recommendations

CD children and their families were counseled by a dietitian regarding a gluten-free diet. They were informed that adherence to a gluten-free diet should be restrictive for the rest of their lives and that a restrictive elimination diet should only be followed by a person diagnosed with CD. It was explained that the rest of the family should continue with a normal (non-restrictive) diet. Families were also explicitly informed about which products they should avoid while on a gluten-free diet and about the possibility of buying gluten-free food and its labeling.

### Microbiota characterization

Following a 2-minute bead-beating step, we extracted DNA from fecal samples using the PureLink Microbiome DNA Purification Kit (Invitrogen, Thermo Fisher, Waltham, MA, USA) according to the manufacturer’s instructions. We then PCR-amplified the variable V4 region of the 16S rRNA gene using 515F-barcoded and 806R-non-barcoded primers (15). We purified amplicons using AMPure XP magnetic beads (Beckman Coulter, Brea, CA, USA) and quantified them using the Picogreen dsDNA quantitation kit (Invitrogen, Thermo Fisher, Waltham, MA, USA). We then pooled equimolar amounts of DNA from individual samples and sequenced the pool on the Illumina MiSeq platform at the Genomic Center at the Bar-Ilan University Azrieli Faculty of Medicine in Tzfat, Israel. Appropriate negative (extraction blanks and PCR blanks) and positive controls (a previously characterized bacteria-rich sample) were included—no contamination was observed in any of the processed gels.

### Microbiota composition comparisons

#### Data processing

FASTQ files were processed using FastQC (version 0.11.5) to perform quality control on the raw sequences. The QIIME2 (16) pipeline (version qiime2-2020.8) was used on 16S rRNA gene raw sequences from microbial communities. The pipeline includes importing the files, demultiplexing using the q2-demux plugin, denoising, dereplication, and removing chimeras using DADA2 algorithm (17), *de novo* amplicon sequence variant (ASV) clustering using vsearch with 97% identity, and assigning taxonomy using the classify-sklearn naïve Bayes classifier against GreenGenes (18) and Silva 138 (19) databases. In total, prior to rarefaction, 340 and 204 ASVs were identified for the family analysis and diet-dynamics analysis, respectively, grouped into 195 and 141 unique taxa. After the QIIME2 pipeline, downstream analysis was performed using the phyloseq (version 1.38.0) R/bioconductor package (20). Taxonomies were cleaned, removing empty taxa. Samples were then rarefied (using the rarefy_even_depth function) to a minimum sequence depth of 4,000 and scaled to relative abundance. Following rarefaction, 339 and 203 ASVs for each analysis, clustered into 194 and 141 unique taxa, remained. Alpha (Faith’s phylogenetic diversity [PD] [21]) and beta diversity (unweighted and weighted UniFrac [22]) were calculated at the ASV level for different groups of samples, as defined below. DESeq2 was used to identify differentially abundant taxa using the best-resolved taxonomy based on the above classification. Plots were generated using ggplot2 (version 3.3.6) (23) and pheatmp (24).

#### Profile comparisons

In this study, we aimed to answer two main questions: (i) How do the microbiota of children with CD differ from their unaffected family members (healthy controls) at time 1, prior to gluten-free diet introduction? (ii) Does exclusion of gluten from the diet affect the CD microbiota?

To answer the first question, we included microbiota data from all family members of the nine recruited families. We compared the alpha diversity of the various family members (CD child, siblings, mother, and father) using a linear mixed model [lmer function in lmerTest R package (25); all model assumptions met] with family ID as a random factor. Similarly, false discovery rate (FDR)-corrected pairwise permutational multivariate analyses of variance (PERMANOVAs) were performed on measures of beta diversity with family ID included as a stratum (random factor). To assess differential taxon abundance, the DESeq2 (version 1.34.0), R/bioconductor package (26) was used. All family members were compared to the CD child group, and significant taxa were identified (adjusted *P*-values <0.05 and |log2foldchange| ≥0.58). Family ID was used as a blocking factor.

To answer the second question, we performed time series analyses for the five children who had fecal samples at all time points (before dietary intervention and following 6 and 12 months of intervention) following quality control filtering. We compared the alpha diversities of samples from the three time points using a repeated-measures analysis of variance (all model assumptions met). Similarly, FDR-corrected pairwise PERMANOVAs were performed on measures of beta diversity with patient ID included as a stratum. DESeq2 was performed, comparing CD child microbiota at the three time points, with patient ID as a blocking factor, and significant taxa were identified (as above).

## RESULTS

### Participant characteristics

The study included nine children with newly diagnosed CD, aged 4.7–10.7 years, and their family members, including 14 siblings aged 1.8–22.6 years (three sisters were over 18 years old and therefore excluded from comparisons of weight and height percentiles) and 17 parents (one mother excluded due to CD diagnosis; mean age of mothers and fathers was 39.3 and 40.5, respectively; Table 1). No statistically significant differences were found in the physical development of CD children and their siblings (up to the age of 18 years), although the number of children with especially short stature (the height <3 percentile) was higher among those with celiac (*n* = 3; 33.3%) than in their siblings (*n* = 1; 9.1%). The percentages of children with normal body weight and height within the 3–97 percentile according to the percentile charts for the population of Polish children (14) in the CD group were 88.9% and 66.7%, respectively, and in the sibling group, they were 90.1% and 90.1%. The body mass index (BMI) of mothers, fathers, and adult siblings ranged from 21.2 to 28.7, 19.7 to 33.2, and 19.5 to 24.4, respectively.

### Celiac disease-associated microbiota

**Fig 1:**
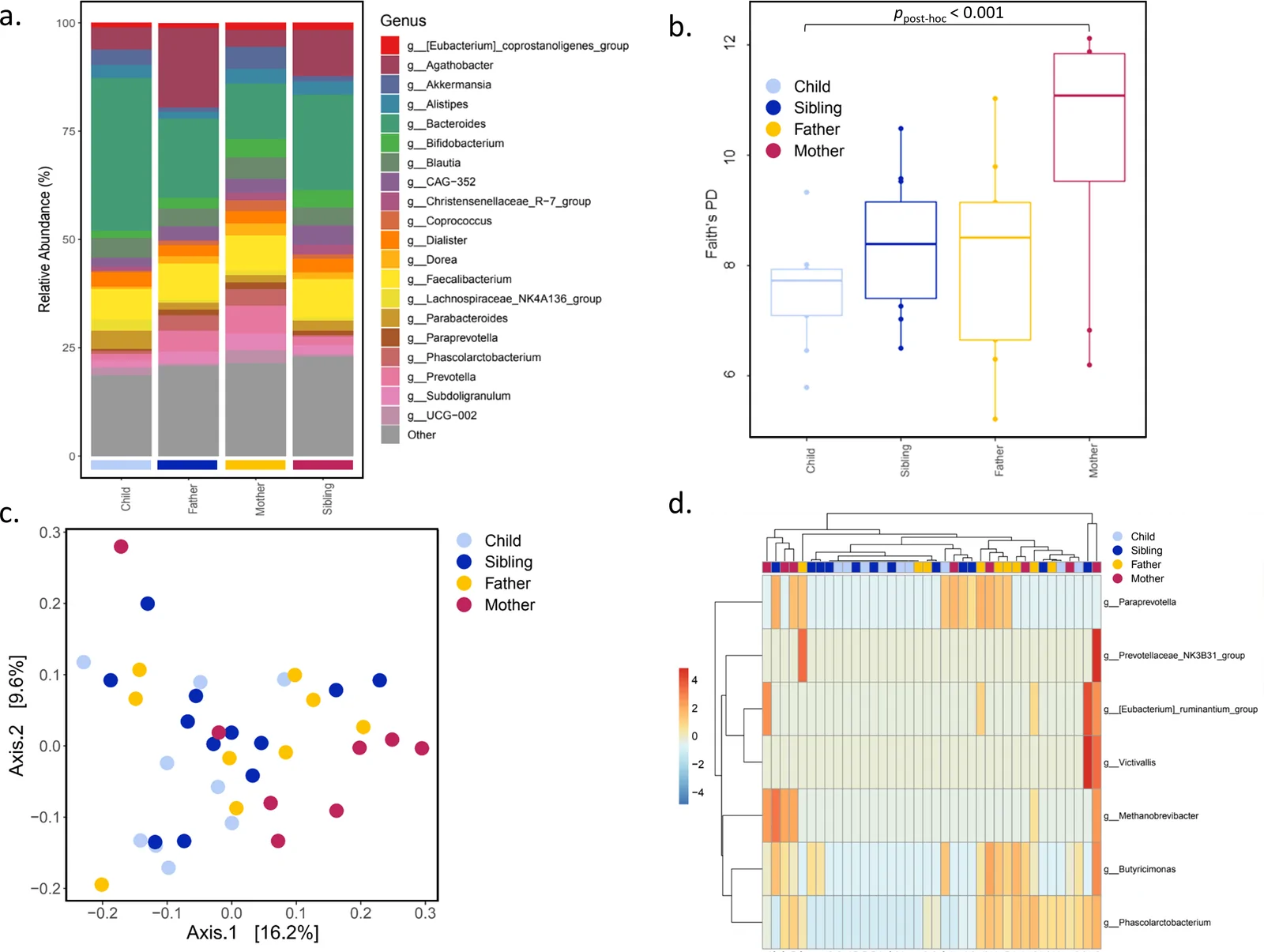
Microbiota profile of children with CD and their unaffected family members. (a) General characterization of the top 20 microbial genera of children with CD and of their unaffected family members (prior to gluten-free diet) (*n* = 9 families). (b) A comparison of the alpha diversity (Faith’s PD) of all family members: mother-child: *P* < 0.001, all other comparisons NS. (c) Principal coordinates analysis (PCoA) ordination of unweighted UniFrac distances. Pairwise PERMANOVAs reveal significant differences (*q* < 0.05) between mother and CD child, father and CD child, mother and father, and mother and unaffected sibling(s). (d) Differential abundance analysis comparing all unaffected family members to CD children did not reveal robust differences in any taxa. The heatbar represents Z-score values: zero is the mean of the row and the other values are the number of standard deviations from the center (*N* = 9).

To determine if there is a specific microbial dysbiosis pattern associated with CD, we characterized (Fig. 1a) and compared nine children with CD to their family members [8 mothers, 9 fathers, and 12 siblings (two did not pass quality control)]. We found a significant effect of family member on alpha diversity, with mothers having significantly richer microbiota than their CD children (*P* < 0.001, Fig. 1b). No differences were found between CD children and siblings without CD. There were significant differences in the community composition of nearly all members, as assessed by PERMANOVA of unweighted UniFrac distances (*P* = 0.002, Fig. 1c): Family member pairs without different microbiota profiles were CD child vs unaffected sibling and father vs sibling (*P*-adjusted pairwise PERMANOVAs with *q* > 0.05). When considering weighted UniFrac, no differences between family members were observed (except a slight trend for mother-CD child: *q* = 0.078). We did not find an effect of sex, age, or BMI (weighted and unweighted UniFrac, not shown). Similarly, including family identifier as a main variable (rather than a random one), stepwise, before the family member comparison did not alter results. Differential abundance analyses of all unaffected family members vs children with CD revealed seven significantly different taxa, but visual examination of a dendrogram of the results did not support family member-wide patterns of differences (Fig. 1d).

### CD diet adherence

**Fig 2:**
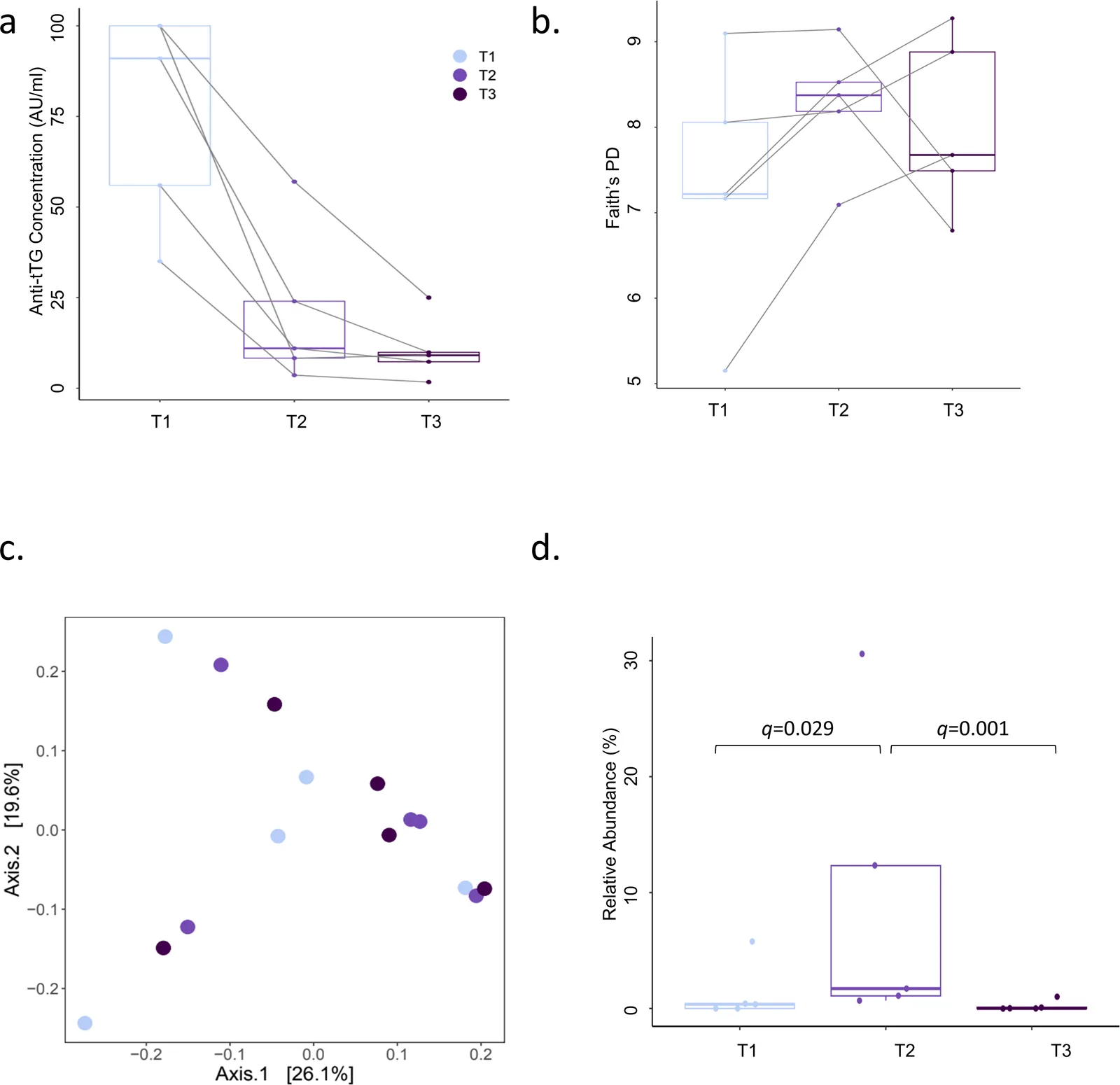
Microbiota dynamics of children with CD following diet intervention (exclusion of gluten). (a) Diet compliance and efficacy were determined by quantifying anti-tTG levels in the blood for all patients who had three fecal samples that passed quality control (*n* = 5). Values of 100 AU/mL are actually “>100 AU/mL,” the maximum reading for this blood test. No changes in (b) alpha diversity (Faith’s PD) or (c) beta diversity (PCoA of unweighted UniFrac distances) were observed throughout the diet intervention. (d) *Akkermansia muciniphila* was significantly more abundant 6 months after diet compared to the other time points.

We monitored adherence to a gluten-free diet by assessing the concentration of anti-tTG antibodies after 6 and 12 months of dietary treatment. Five of the nine patients followed a gluten-free diet and provided fecal samples at all three time points that could be successfully processed for microbiota characterization. In all but one of the five cases, anti-tTG concentrations normalized to <10 AU/mL at the 12 month follow-up (Fig. 2a; Table S1). In the final case, the antibody concentration was 25 AU/mL; however, the initial anti-tTG levels in this patient were very high (>100 AU/mL), which suggests that despite the lack of normalization, this child also complied to a gluten-free diet.

### Effects of diet modification on CD children’s microbiota

To understand if gluten-free diet in CD patients is associated with microbial shifts, we followed five children through 1 year of diet intervention characterizing and comparing their microbiota profiles prior to intervention, 6 months into intervention, and after 1 year of intervention. Interestingly, no significant differences were found in alpha diversity over the course of the gluten-free diet intervention (*P* = 0.314, Fig. 2b), and only a trend toward significant differences was observed between the microbiota pre-intervention and following one year of compliance (*P*-adjusted pairwise PERMANOVAs: *q* = 0.063 for T1 vs T2 and T1 vs T3, Fig. 2c). When examining the alpha diversity dynamics visually, though, we did notice two divergent patterns. Just over half of the patients had increasing diversity throughout the period, while the other half showed a steep decrease in the latter portion of the follow-up. No demographic differences were identified between the subgroups that could explain these findings (Table S1). The only significant difference in taxa abundances between time points was in *Akkermansia muciniphila* which was significantly increased in T2 (6 months of diet) relative to pre-diet microbiota (DESeq2: *q* = 0.029) and samples from T3 (1 year of diet; DESeq: *q* = 0.001, Fig. 2d).

## DISCUSSION

Here, we examined the microbiota of children with CD compared to their unaffected family members. We did not find differences in the microbiota of siblings with and without CD despite a wealth of evidence in the literature supporting CD-specific microbiota, though these studies did not use siblings as controls [e.g., reference (27) and reviewed in reference (10)]. Interestingly, there is also evidence supporting our findings. A study by Zafeiropoulou et al. (28) did not find evidence of a CD microbiota when comparing CD patients to both healthy siblings and unrelated healthy controls. Having a first-degree relative with celiac, here, the CD child can signify increased risk of developing CD for other family members because of shared genetic susceptibility. The prevalence of CD in first-degree relatives ranges from 1.6% to 38% (29). With our family-wide CD screening, we diagnosed CD in one mother (1:32; 3.1%, removed from analyses) in addition to the nine focal children. The absence of (weighted UniFrac, DESeq2, child-sibling comparisons) or minimal (between mother and child) differences observed between children diagnosed with celiac and their siblings or other family members is puzzling. Many CD cases, though, are only diagnosed later in life (30) suggesting that some of the unaffected family members may be living in a pre-CD state, below clinical detection. For example, while Bodkhe et al. (31) found some differences between CD patients and their family members, there were shared differences between these groups and healthy controls, further supporting the hypothesis of a pre-CD microbiota. There is also evidence that environmental factors can have strong or even stronger effects than disease state on the microbiota. For example, dusts and other microbes in the built environment can affect the microbiome, as can exposure to smoke and toxins, living with pets, shared diets, and even socioeconomic status (32). Furthermore, there is accumulating evidence of the effects of host genetics on microbiota (33). Here, we may be observing consequences of a genetics-diet-household environment dynamics in which the disease effects are masked by those of the shared family traits, but based on unweighted UniFrac evidence (both family identifier and family position were significant), weighted UniFrac evidence (neither family identifier nor family position was significant), and the dendrogram of differentially abundant taxa (Fig. 1d), this does not seem to be the case.

Interestingly, transition to a gluten-free diet also did not greatly affect the microbiota among children followed for 1 year post diet control. Diet adherence was confirmed in all children with anti-tTG antibody profiling but shifts in microbiota composition, assessed by unweighted UniFrac, were only marginally significant—though this could be a byproduct of the limited sample size. We did, however, observe an increase in *Akkermansia muciniphila* 6 months into diet intervention. *Akkermansia* is associated with healthy gut function and was found to be increased in non-CD controls (31, 34) in the past, suggesting at least a short-term protective effect of diet. This increase, though, was not preserved to the final time point, which could suggest a peak microbial contribution to CD mediation which tapers with sustained diet control. Lack of broad microbiota shifts is surprising because one of the main factors known to shape the gut microbiota, regardless of disease status, is host diet (35). Like above, though, the environment-based microbiota (family members’ non-gluten-free habits, shared environment, genetics) may have led to decreased microbiota shifts in CD children. While a slew of studies found evidence for microbial shifts following gluten-free diet both in the context of CD (34, 36) and in its absence (37), there is also some evidence that CD diet control does not completely restore the microbiota to that of healthy controls, though in some contexts, some shifts away from CD-associated dysbiosis have been recorded (38). A study comparing CD patients with strict gluten-free diet adherence (and TG control) to those with weak adherence (no TG control) did not find differences between the groups’ microbiota profiles either (39). We may not have found shifts over time if the change in diet was not so drastic in practice, but based on our data, there was normalization of anti-tTG concentrations following diet. Furthermore, based on another study in Poland, adherence to a gluten-free diet in patients with CD is high, especially in children. In a survey in a group of 224 children with CD, 98.7% of respondents declared adherence to a gluten-free diet (40).

On the whole, we provide evidence that CD is not consistently associated with microbiota dysbiosis. This could be a result of controlling for confounding factors (within-family comparisons) that may differ between patient and control populations or may be a result of the effect of host genetics on microbiota (41, 42). The family-focused model may even result in a masking effect as there is also support for familial similarity of the gut microbiota (43). Diet control did not change the microbiota in this small Polish cohort of CD patients. This puzzling finding could be a technical shortcoming (small sample size, sparse temporal sampling, the low resolution of 16S amplicon sequencing data) or a biological one [wide range of ages, though all CD patients were over 4 years old, an age at which the microbiota is considered to be rather stable and adult-like (44)] but is worth further exploration. Future research in a similar family-focused manner or for well-matched, unrelated pairs of cases and controls may shed light on how important the microbiota really is in this disease’s pathology and management.

## Data Availability

Data are available in the EBI database under project identifier PRJEB63123.

## SUPPLEMENTAL MATERIAL

Supplemental Table S1 spectrum.01463-23-s0001.xlsx Patient characteristics of the five patients sampled longitudinally throughout gluten-free diet intervention.

ASM does not own the copyrights to Supplemental Material that may be linked to, or accessed through, an article. The authors have granted ASM a non-exclusive, world-wide license to publish the Supplemental Material files. Please contact the corresponding author directly for reuse.
